# Human Macrophages Exhibit GM-CSF Dependent Restriction of *Mycobacterium tuberculosis* Infection *via* Regulating Their Self-Survival, Differentiation and Metabolism

**DOI:** 10.3389/fimmu.2022.859116

**Published:** 2022-05-12

**Authors:** Abhishek Mishra, Vipul K. Singh, Chinnaswamy Jagannath, Selvakumar Subbian, Blanca I. Restrepo, Marie-Claire Gauduin, Arshad Khan

**Affiliations:** ^1^ Department of Pathology and Genomic Medicine, Houston Methodist Research Institute, Houston, TX, United States; ^2^ Department of Medicine, New Jersey Medical School, Public Health Research Institute, Newark, NJ, United States; ^3^ University of Texas School of Public Health, Brownsville, TX, United States; ^4^ Disease Intervention and Prevention, Texas Biomedical Research Institute, San Antonio, TX, United States

**Keywords:** macrophages, *Mycobacterium tuberculosis*, tuberculosis, granulocyte macrophage colony-stimulating factor, cell death, antigen presentation, autophagy, cellular metabolism

## Abstract

GM-CSF is an important cytokine that regulates the proliferation of monocytes/macrophages and its various functions during health and disease. Although growing evidences support the notion that GM-CSF could play a major role in immunity against tuberculosis (TB) infection, the mechanism of GM-CSF mediated protective effect against TB remains largely unknown. Here in this study we examined the secreted levels of GM-CSF by human macrophages from different donors along with the GM-CSF dependent cellular processes that are critical for control of *M. tuberculosis* infection. While macrophage of different donors varied in their ability to produce GM-CSF, a significant correlation was observed between secreted levels of GM-CSF, survial of macrophages and intra-macrophage control of *Mycobacterium tuberculosis* bacilli. GM-CSF levels secreted by macrophages negatively correlated with the intra-macrophage *M. tuberculosis* burden, survival of infected host macrophages positively correlated with their GM-CSF levels. GM-CSF-dependent prolonged survival of human macrophages also correlated with significantly decreased bacterial burden and increased expression of self-renewal/cell-survival associated genes such as *BCL-2* and *HSP27*. Antibody-mediated depletion of GM-CSF in macrophages resulted in induction of significantly elevated levels of apoptotic/necrotic cell death and a simultaneous decrease in autophagic flux. Additionally, protective macrophages against *M. tuberculosis* that produced more GM-CSF, induced a stronger granulomatous response and produced significantly increased levels of IL-1β, IL-12 and IL-10 and decreased levels of TNF-α and IL-6. In parallel, macrophages isolated from the peripheral blood of active TB patients exhibited reduced capacity to control the intracellular growth of *M. tuberculosis* and produced significantly lower levels of GM-CSF. Remarkably, as compared to healthy controls, macrophages of active TB patients exhibited significantly altered metabolic state correlating with their GM-CSF secretion levels. Altogether, these results suggest that relative levels of GM-CSF produced by human macrophages plays a critical role in preventing cell death and maintaining a protective differentiation and metabolic state of the host cell against *M. tuberculosis* infection.

## Introduction

Tuberculosis (TB) remains a major health concern worldwide, despite vaccine coverage efforts. With a current worldwide incidence of approximately 10 million new TB cases and 1.3 million associated deaths annually, TB is the leading cause of death by an infectious disease worldwide (1). TB also accounts for approximately 40% of deaths among people with human immunodeficiency virus (HIV) (2). Failure to control TB is attributed to several factors, including treatment non-compliance and failure, emergence of multi drug resistant (MDR) strains, poor diagnostic tools, poor vaccine efficacy of BCG, and socioeconomic challenges (3). A vaccine that effectively protects against different forms of TB across all age groups would dramatically reduce disease burden worldwide. However, identifying key immune components that protect against TB remains a major challenge in developing an effective vaccine.

Traditionally, protective immunity to TB has been attributed to a T-cell-mediated immune response, with CD4+ T cells playing a critical role (4, 5). However, more recent experimental evidence now suggests that host resistance to *Mycobacterium tuberculosis* infection could be independent of IFN-γ and TNF-α secretion from CD4+ T cells (6, 7). Evidence from clinical and experimental studies supports a more critical role of innate cells in protective immunity against TB (8, 9). Macrophages, the primary innate cell involved in the initial uptake of *M. tuberculosis*, also possess T-cell-independent, intrinsic bactericidal activity (10, 11). Further, the relative permissiveness of macrophages for intracellular growth of *M. tuberculosis* varies, supporting the relevance of macrophage-mediated innate immunity in TB disease (12). However, the mechanism(s) through which macrophages restrict intra-macrophage growth of *M. tuberculosis* to provide protective immunity against TB is rather unclear. Granulocyte-macrophage colony-stimulating factor (GM-CSF) is one key component that is now being increasingly recognized for its critical role in resistance against TB (13–15). GM-CSF could be produced by a variety of cells including, conventional and non-conventional T cells, macrophages, alveolar epithelial cells; and importance of this growth factor in *M. tuberculosis* infection control has previously been reported by us and others albeit in a mouse model of tuberculosis which is inherently susceptible to tuberculosis (15, 16). In a more recent study we found that human macrophages produce significantly higher amounts of GM-CSF than mouse macrophages, and have significantly increased cell survival and *M. tuberculosis* infection control abilities (17). This finding led us to hypothesize that lower levels of GM-CSF may relate to TB susceptibility in humans.

Here in this study we quantified the levels of GM-CSF produced by primary macrophages isolated from active TB and healthy donors and examined the ability of the cells to control *M. tuberculosis* infection *in vitro*. The amount of GM-CSF produced by macrophages varied significantly between donors, but we observed a clear correlation between the secreted levels of GM-CSF and the ability of macrophages to prolong their survival and control *M. tuberculosis* infection. In macrophages derived from active TB patients, we observed lower levels of GM-CSF production, reduced self-survival, and increased proliferation of *M. tuberculosis* bacilli. We interrogated the cellular mechanisms underlying GM-CSF-mediated TB immunity using antibody-mediated GM-CSF blockade and exogenous GM-CSF supplementation in primary human macrophages. We assessed the effect of neutralized and enhanced GM-CSF signaling on host macrophages survival, bacterial burden, differentiation state, inflammation state, autophagy flux, phagosome maturation events, antigen processing, cytokine secretion, and metabolism. Our findings indicate that self-survival, anti-inflammatory properties, metabolic state, differentiation state, and autophagic processes of macrophages are directly related to GM-CSF-dependent intracellular restriction of *M. tuberculosis*. These data emphasize the importance of macrophage produced GM-CSF in controlling *M. tuberculosis* infection and define GM-CSF-dependent cellular pathways that contribute to intracellular defense mechanisms against TB.

## Methods

### Monocytes/Macrophages From Healthy Donors, Contacts, or TB Patients

All blood samples were collected per approved institutional review board protocols. CD14 magnetic beads (Miltenyi Inc., USA) were used to purify monocytes from PBMCs that were plated in 6 or 24 tissue culture well plates at a density of 5x10^6^ and 1x10^6^ cells per well, respectively. Eight-well slide chambers or coverslips received 10^4^ cells per chamber for confocal/immunofluorescent imaging studies. CD14 bead purified monocytes were grown in Iscove’s medium (IMDM) with 10% fetal bovine serum (FBS) and 10 μg/mL penicillin and gentamycin for 24 hours and then plated in antibiotics-free medium until 7 days for before differentiation into macrophages.

### Human TB Patients and Healthy Contacts

PBMCs were collected from deidentified, known TB patients and their healthy contacts under the approved IRB protocols of Dr. Restrepo from Reynosa, Mexico, under a collaboration. Approved IRB protocol: HSC-SPH-12-0037.

#### Bacterial Strains, Culture Conditions, and Infection of macrophages

Log phase organisms of wild-type *M. tuberculosis H37Rv* (ATCC 27294) were cultured in Middlebrook 7H9 broth for 7 days and were then frozen in aliquots. Before use, aliquots were thawed, washed three times in PBS (12,000 rpm; 15 mins), sonicated at 4 watts using a sonication probe and dispersed suspension matched with McFarland #1 in turbidity (10^8^ CFU/mL). Macrophages were infected with *M. tuberculosis* at MOI of 1 with a 4 hour phagocytosis incubation time. Extracellular bacteria were then removed through washing with sterile IMDM medium thrice and macrophages were further incubated at 37°C in a CO_2_ incubator.

### Cytokine and Cathilicidin ELISA Assays

Cell supernatants were tested for cytokines using sandwich ELISA kits (Biolegend and R&D systems, USA) for various cytokines (TNF-α, IL-1β, IL-4, IL-6, IL-10, IL-12, and IL-13) secreted by GM-CSF competent and GM-CSF depleted macrophages after infection with *M. tuberculosis*. For the quantification of Cathilicidin through ELISA (Novus Biologicals), cell lysates of GM-CSF competent and GM-CSF depleted macrophages after infection with *M. tuberculosis* were used.

### *M. tuberculosis* Growth Assays in Macrophages, Cell Viability Assays, and Cell Death Assays

Macrophages were lysed with 0.05% SDS at different time points post-*M. tuberculosis* infection with or without hGM-CSF supplementation, α-hGM-CSF antibodies, or other pharmacological agents/inhibitors. Cell culture media along with hGM-CSF and α-hGM-CSF antibodies were replaced every alternate day during these experiments and assays. At different time points, lysates were plated at serial 10-fold dilutions in PBS using 7H11 Middlebrook agar plates (Difco Laboratories, Surrey, UK). The plates were incubated at 37°C for 3 weeks before counting CFUs. Data were expressed as log10- CFUs per million macrophages. Alamar Blue cell viability reagent (Life Technologies, DAL1025) was used to assess cell viability by adding the ready-to-use 1X solution to uninfected or *M. tuberculosis*-infected macrophages at various time points, followed by fluorescence measurement through fluorimeter per the manufacturer’s protocol. A fluorescence-based apoptosis/necrosis detection kit (Abcam #ab176749) that can simultaneously monitor apoptotic, necrotic, and healthy cells was used to measure and quantify apoptosis and necrosis in *M. tuberculosis* infected, uninfected, differentiated, and undifferentiated macrophages using a fluorescence microscope.

### *Ex Vivo M. tuberculosis* Ag85B Antigen Presentation to CD4 T Cells

We have previously described this method in detail, and the original method described by the Harding lab has been extensively used by us and others for *in vitro* antigen presentation by macrophages (18). Briefly, *M. tuberculosis*-infected macrophages were washed after a 4-hour infection and overlaid with the F9A6-CD4 T cell hybridoma (gift from Dr. David Canaday lab) that recognizes an Ag85B epitope in the context of human HLA-DR1. IL-2 secreted from hybridoma T cells or other cytokines secreted from *M. tuberculosis*-infected macrophages were determined using a sandwich ELISA kit (Biolegend).

### Western Blot Analysis

Six well tissue culture plates were seeded with macrophages. They were infected with *M. tuberculosis* for 4 hours at MOI of 1 and then washed three times with PBS and re-plated in the medium. At different time points, MΦs were washed three times with 1x PBS, and 50 µL RIPA buffer containing APM (anti-protease mix) was added to each well and incubated for 15 minutes. Lysates were then collected, and protein quantification (Bradford assay, Pierce Coomassie Plus cat no. 23238; Thermo Fisher Scientific) was performed. The quantitative Wes capillary immunoassay was used for the Western blot, in which the lysates were separated and detected using Wes separation capillary cartridge 12-230 kDa along with Wes Anti-Rabbit Detection Module (Simple Western system and Compass Software, Protein Simple). In brief, glass microcapillaries were loaded with stacking and separation matrices followed by sample loading. During capillary electrophoresis, proteins were separated by size and then immobilized to the capillary wall (19). Samples were loaded at 1 mg/ml dilution and the primary rabbit antibody ATG 5 and 7 was used at a dilution of 1:50 (ATG5: cat no.2360;cell signaling and ATG7: cat no.8558) and β-actin (Rabbit monoclonal, Sigma-Aldrich # SAB5600204) were used at 1:50 dilution. Data were analyzed with the Compass software (version 2.6.7). The area under the curve (AUC), which represents the signal intensity of the chemiluminescent reaction was analyzed for all the antibodies and β-actin. Values given for protein expression were normalized to β-actin. Quantitation of protein levels (area under each peak; arbitrary units [A.U.]) were performed using the Compass software (version 2.6.7).

### Evaluation of Autophagosome Puncta, LC3B Containing Endosomes, and Their Localization With rfp *M. tuberculosis* Within Macrophages

GM-CSF competent and depleted macrophages were plated in 8-well chamber slides at a density of 10^4^ cells per chamber and infected with red fluorescent protein (RFP) expressing *M. tuberculosis* (*rfp M. tuberculosis*) at an MOI of 1. Per established procedures, autophagy was evaluated using at least three criteria. Fixed macrophages were permeabilized and stained using specific antibodies against LC3 followed by secondary staining with Alexfluor488 (Jackson Immunoresearch # 109-545-003) as described previously (19). Percent *rfp M. tuberculosis* phagosomes colocalizing with antibodies was conducted as described previously (20).

### Quantitative (QPCR) Analysis

Total RNA from macrophages was extracted using RNAeasy mini kit (Qiagen, Germany). RNA concentration and purity ratios (OD260/280, OD260/230) were measured using the NanoDrop ND-1000 spectrophotometer (Thermo Fisher Scientific, USA). cDNA synthesis was performed on a CFX96 Real-Time PCR System (Biorad, USA) using the 2X OneStep qRT-PCR Mastermix Kit (Applied Biosystems, USA) according to manufacturer’s instruction. Quantitative PCR (qPCR) was performed using SYBR green probe and gene specific primers (**Supplementary Table S2**). Threshold cycle numbers were transformed to ΔΔCt values, and the results were expressed relative to the reference gene, GAPDH. Gene expression data was performed using GraphPad Prism ver. 6.0 suite (GraphPad Software). Student’s t-test was used for mean comparison between GM-CSF competent and depleted macrophages. Significance was set at the 0.05 level.

### Nitric Oxide (NO) and Reactive Oxygen Specice (ROS) Estimation

Macrophages were plated in 96-well plates at a density of 1x10^4^ cells per well in triplicate and infected with *M. tuberculosis* (MOI=1). Cells were then treated with fluorescent probes for the quantification of NO using diaminofluorescein diacetate (DAF-2 DA), per manufacturer’s instructions (Enzo Life Sciences, USA); DCFDA was used to detect ROS. Quantification of NO/ROS release was determined by calculating the fluorescence emitted over time using an ^ex^485 nm/^em^515 nm and plotting AFUs (± SD) against time using Ascent fluoroscan software version 2.6.

### Statistical Analysis

All experiments were performed at least 3 times containing triplicate wells per experimental group or combination. Statistical parameters including the definition of central value and spread (mean ± SD) of macrophages per group are annotated in the corresponding figure legends. Statistical analysis was performed with GraphPad Prism version by using, unless otherwise stated, unpaired t tests with 95% confidence intervals. P values for CFU count in macrophages were determined using 1- or 2-way ANOVA with Tukey’s posthoc test (GraphPad PRISM software).

## Results

### Monocyte GM-CSF Levels Correlate With Host Cell Survival and Intracellular *M. tuberculosis* Proliferation

**Figure 1:**
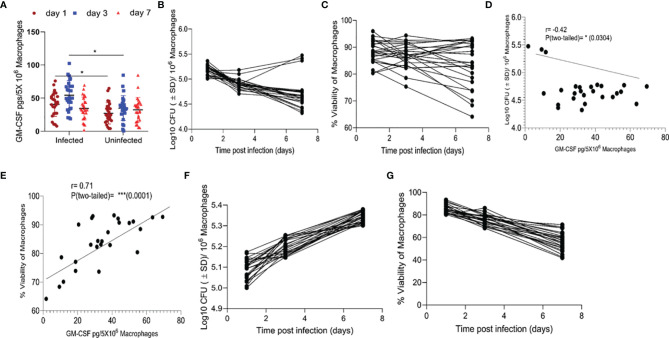
Association between GM-CSF secretion levels, bacterial burden, and cell viability of macrophages post-*M. tuberculosis* infection. **(A)** Secreted levels of GM-CSF by *M. tuberculosis* in infected and uninfected macrophages of different individuals after differentiation.pg = picogram. **(B)** *M. tuberculosis* burden in macrophages of different individuals after differentiation as measured by CFU assay over seven days of infection. **(C)** Cell viability of *M. tuberculosis*-infected macrophages after differentiation measured by alamarBlue assay over seven days of infection. **(D)** Two-tailed correlation analysis between secreted GM-CSF levels and *M. tuberculosis* burden in macrophages on day 7 post-infection. **(E)** Two-tailed correlation analysis between secreted GM-CSF levels and cell viability of macrophages on day 7 post-infection. **(F)** *M. tuberculosis* burden in undifferentiated macrophages by CFU assay over 7 days of infection. **(G)** Cell viability of *M. tuberculosis*-infected undifferentiated macrophages by alamarBlue assay over seven days of infection. Data represent average of three independent experiments carried out in duplicate. Bars and error bars represent mean and SD, respectively. *p ≤ 0.05, 0.005, ***p ≤ 0.0005.

We compared the levels of GM-CSF produced by monocytes isolated from the peripheral blood of various healthy subjects (HS) (n=26) and analyzed their bacterial load and survival during *M. tuberculosis* infection *in vitro*. Prior to *M. tuberculosis* infection, we allowed their differentiation into macrophages and measured GM-CSF production. CD14+ classic monocytes, isolated from the peripheral blood of different HS were differentiated *via* adherence on cell culture plates. Quantifiable levels of GM-CSF in cell culture supernatants were not detectable before 48 h using the Sandwich ELISA method (**Supplementary Figure S1**). After seven days of differentiation, adherent, fully differentiated macrophages were isolated and infected with *M. tuberculosis* at a low multiplicity of infection (MOI) of one. GM-CSF levels were immediately detectable post-infection in these differentiated cells, though they varied significantly over time between donors (**Figure 1A**). We also measured GM-CSF levels in an additional, uninfected set of differentiated macrophages for comparison with infected cells from the same donors set. Relative to uninfected cells, infected cells produced significantly more GM-CSF early after infection (1-3 days) in most of the HS. However, GM-CSF levels became more similar between these two groups at three days post infection. We compared intracellular colony-forming units (CFUs) of *M. tuberculosis*, macrophage viability, and GM-CSF levels over time after infection and found that intracellular CFUs declined in most individuals, though three individuals (11.5%) showed an increase in intracellular *M. tuberculosis* burden (**Figure 1B**). Similarly, *M. tuberculosis*-infected macrophage viability declined slowly but remained over 80% seven days post-infection in a majority of individuals (77%) (**Figure 1C**, **Supplementary Table 1**). Macrophages from six individuals (23%) lost over 20% cell viability by seven days post-infection and exhibited accelerated cell death following infection (**Figure 1C**). Increased production of GM-CSF directly correlated with prolonged survival and improved intracellular bacterial control of macrophages (**Figures 1D, E**). GM-CSF levels and bacterial burden were inversely correlated in macrophages (**Figure 1D**). On the other hand, there was a strong positive correlation between GM-CSF levels and macrophage viability after infection (**Figure 1E**) that was also observed in uninfected cells (**Supplementary Figure S2**). Thus, GM-CSF may contribute to long-term survival in macrophages while preventing *M. tuberculosis*-induced cell death. To investigate the association between macrophage GM-CSF production, cell survival, and *M. tuberculosis* infection control in undifferentiated cells, we infected CD14+ monocytes from different donors prior to differentiation (**Figures 1F, G**). Undifferentiated monocytes did not produce detectable levels of GM-CSF following infection with *M. tuberculosis* (data not shown), and an increase in bacterial burden was observed in these cells (**Figure 1F**). Furthermore, viability of undifferentiated monocytes decreased rapidly relative to differentiated macrophages over the seven-day infection period (**Figure 1G**). These data suggest that undifferentiated monocytes are more permissive for *M. tuberculosis* growth and infection-induced cell death as compared to differentiated macrophages.

### Host Macrophage Survival and Intracellular *M. tuberculosis* Burden Is Dependent on GM-CSF Signaling

**Figure 2:**
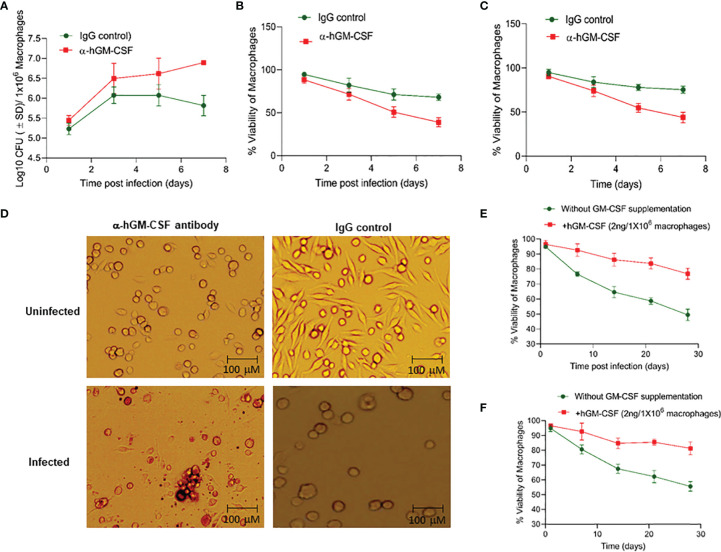
GM-CSF-dependent control of bacterial burden, cell survival, and differentiation of human macrophages during *M. tuberculosis* infection. **(A)** *M. tuberculosis* burden in α-hGM-CSF antibody-treated undifferentiated human macrophages versus IgG isotype-treated undifferentiated macrophages as measured through CFU assay over seven days of infection. Concentration of α-hGM-CSF antibody/IgG isotype used for treatment was 2 μg/10^6^ macrophages. **(B)** Cell viability of *M. tuberculosis*-infected and α-hGM-CSF antibody-treated undifferentiated human macrophages versus IgG isotype-treated undifferentiated macrophages measured by alamarBlue assay over seven days of infection. **(C)** Cell viability of α-hGM-CSF antibody-treated undifferentiated human macrophages versus IgG isotype-treated undifferentiated macrophages measured by alamarBlue assay over seven days. **(D)** Morphology of α-hGM-CSF antibody-treated macrophages versus untreated macrophages with and without *M. tuberculosis* infection at day 7 post-infection/treatment. Images were acquired at 20x magnification with an inverted brightfield microscope using macrophages of a single random healthy donor. **(E)** Cell viability of -infected and hGM-CSF-treated undifferentiated macrophages versus untreated undifferentiated macrophages measured by alamarBlue assay over seven days of infection. **(F)** Cell viability of uninfected hGM-CSF-treated undifferentiated macrophages versus untreated undifferentiated macrophages measured by alamarBlue assay over seven days of infection. Data represent average of three independent experiments carried out in duplicate. Bars and error bars represent means and SD, respectively.

To determine whether macrophage permissiveness for *M. tuberculosis* growth and infection-induced cell death were dependent on GM-CSF, we assessed *M. tuberculosis* proliferation in healthy and infected macrophages in the presence and absence of GM-CSF. We used an anti-human GM-CSF antibody (α-hGM-CSF) to neutralize GM-CSF effect on macrophages. We added α-hGM-CSF antibodies (2 μg/1 million cells) to monocytes before or after differentiation, as earlier described. *M. tuberculosis-*infected undifferentiated monocytes treated with α-hGM-CSF showed increased bacterial burden and decreased survival over a period of seven days relative to those treated with IgG isotype (**Figures 2A, B**). Uninfected and undifferentiated monocytes treated with α-hGM-CSF antibody had significantly reduced survival over a period of seven days relative to untreated controls (**Figure 2C**). Microscopic examination revealed inhibition of cell differentiation in uninfected and infected monocytes treated with α-hGM-CSF (**Figure 2D**). Similar results were also observed when hGM-CSF receptor (CSF2RA) was blocked through anti-CD116 antibody (Data not shown).

We next assessed whether exogenous hGM-CSF enhanced monocyte survival during differentiation in the presence and absence of *M. tuberculosis* infection. We added a range of hGM-CSF doses (1-10 ng/5 million cells) to undifferentiated monocytes isolated from three human subjects. Survival in infected and uninfected controls was assessed in cells from the same donor in the presence or absence of exogenous hGM-CSF. hGM-CSF (2 ng/1 million cells) enhanced cell survival by over 30% by day 28 post-*M. tuberculosis* infection relative to untreated infected macrophages (**Figure 2E**). Doses exceeding 2 ng hGM-CSF/1 million cells enhanced cell survival but also induced cell proliferation and inhibited differentiation (data not shown). Uninfected monocytes treated with 2 ng hGM-CSF/1 million monocytes survived significantly longer relative to untreated monocytes (**Figure 2F**). These data suggest that a limited supplementation of GM-CSF could further extend the survival of monocyte during *M. tuberculosis* infection.

**Figure 3:**
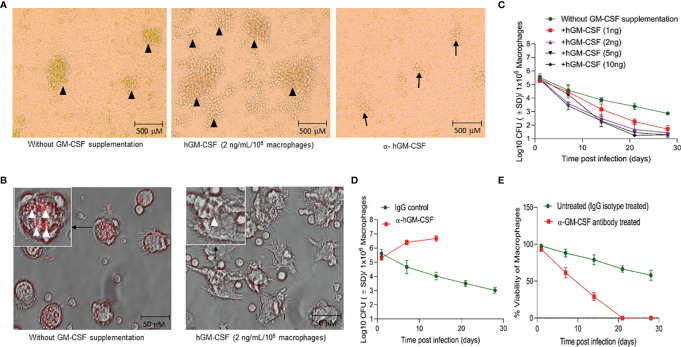
GM-CSF induces formation of granulomatous structures and differentiation of macrophages to control bacterial burden and host cell survival during *M. tuberculosis* infection. **(A)** *In vitro* granuloma-like structure formation in untreated, hGM-CSF (2 ng/10^6^macrophages), α-hGM-CSF (2 μg/10^6^ macrophages) treated macrophages. Macrophages were infected with *M. tuberculosis* (H37Rv; MOI 1:1) resulting in the formation of granulomas by day 7 post-infection. Images were acquired at 4x magnification with an inverted brightfield microscope using macrophages of a single random healthy donor. **(B)** At day 14 post-infection with *M. tuberculosis* (rfpH37Rv; MOI 1:1), untreated cells show undifferentiated macrophage-like morphology, including rounder shape and shorter microvilli, whereas hGM-CSF (2 ng/10^6^ macrophages)-treated cells exhibit morphology similar to differentiated macrophages with flat, elongated shape and longer dendrons. Images were acquired at 40x magnification with an inverted fluorescent/brightfield microscope using macrophages of a single random healthy donor. More *M. tuberculosis* bacilli (red) are visible in untreated cells relative to hGM-CSF-treated (2 ng/10^6^ macrophages) cells. Images in A and B are representative images from twelve experiments with 4X and 40X magnification, respectively. **(C)** Dose-dependent effect of hGM-CSF treatment on bacterial burden within macrophages up to 28 days post-*M. tuberculosis* infection (H37Rv; MOI 1:1). **(D)** Effect of α-hGM-CSF antibody treatment on bacterial burden within macrophages up to 28 days post-*M. tuberculosis* infection (H37Rv; MOI 1:1). Bacterial burden was not determined in α-hGM-CSF treated macrophages because of excessive cell death in this group after 14 days post-infection. **(E)** Effect of α-hGM-CSF antibody treatment on cell viability of macrophages up to 28 days post-*M. tuberculosis* infection (H37Rv; MOI 1:1). Treatment with hGM-CSF or α-GM-CSF antibody/IgG isotype (untreated control) was done every seven days starting from the day of infection. Data represent average of three independent experiments carried out in duplicate. Bars and error bars represent means and SD, respectively.

Exogenous hGM-CSF also increased the formation of granulomatous structures in peripheral blood mononuclear cells (PBMC)-derived monocytes (**Figure 3A**), a previously documented phenomenon (21). While *M. tuberculosis* infection itself induced granuloma-like structures in macrophage culture, exogenous hGM-CSF further increased the size and number of granuloma-like centers (**Figure 3A**). Conversely, treatment with α-hGM-CSF reduced the granulomatous response in macrophages and significantly reduced the number and the size of granulomatous structures. These granuloma-like structures were not seen in uninfected macrophages (data not shown). Granuloma-like structures began to disappear 14 days post-infection/exogenous hGM-CSF treatment, but the number of *M. tuberculosis* CFUs remained significantly low relative to untreated macrophages (**Figures 3B, C**). With increasing exogenous hGM-CSF doses, a time-dependent decrease in intracellular CFUs was observed, indicating that GM-CSF may restrict *M. tuberculosis* by promoting granulomatous response in cells (**Figure 3C**). Relative to untreated cells, treatment with 2 ng of hGM-CSF/1 million macrophages reduced bacterial burden by over 2.5 logs by day 28 post-infection. Notably, granuloma-like structures were also seen early during infection (5-10 days) in untreated macrophages, and though these cells could prevent intracellular proliferation of *M. tuberculosis* bacilli, they could not reduce bacterial burden with time (**Figures 3A–C**). We next examined if intrinsic levels of GM-CSF produced by macrophages could prevent intracellular proliferation of *M. tuberculosis*. The endocrine effect of GM-CSF in macrophages was blocked using α-hGM-CSF, and intra-macrophage CFUs were examined up to 28 days post-infection (**Figure 3D**). A marked increase in *M. tuberculosis* CFUs was observed in the first 14 days relative to untreated macrophages. After 21 days 100% cell death was observed in α-hGM-CSF treated macrophages whereas untreated macrophages maintained more than 50% viability at this time point (**Figure 3E**). Taken together, these results clearly indicated that self-survival of macrophages as well as their ability to form granuloma and contain *M. tuberculosis* infection is dependent on GM-CSF.

### GM-CSF Mediated Cell-Survival and Antimicrobial Effector Functions Are Partly Autophagy-Dependent

**Figure 4:**
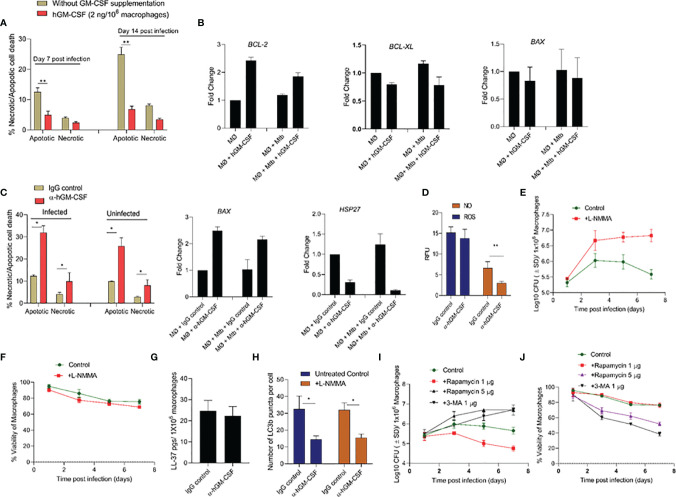
GM-CSF regulates apoptotic/necrotic cell death and bacterial control through autophagy-dependent and independent pathways. **(A)** Percentage and proportion of apoptotic and necrotic cell death after 7 and 14 days post-*M. tuberculosis* infection in untreated and hGM-CSF(2 ng/10^6^ macrophages)-treated macrophages. At 7 and 14 days post-*M. tuberculosis* infection, hGM-CSF-treated and untreated macrophages were incubated with Apopxin solution and 7-ADD, and fluorescence [Ex/Em=490/525 nm (apoptosis, Ex/Em=550/650 nm (necrosis), Ex/Em=405/450 nm (healthy cells)] was measured by fluorescence microscopy at a magnification of 20X counting 25 fields per replicate for each condition. **(B)** Transcription level expression of cell survival cell death-associated genes *BCL-2, BCL-XL*, and *BAX* in hGM-CSF (2 ng/10^6^ macrophages)-treated and untreated macrophages with and without *M. tuberculosis* infection. Gene expression was measured at seven days post-treatment/infection *via* qPCR assay. **(C)** Percentage and proportion of apoptotic and necrotic cell death in α-hGM-CSF antibody-treated and IgG isotype-treated macrophages with and without *M. tuberculosis* infection at day 7. Fluorescent microscopy-based necrosis/apoptosis cell death assay kit was used to determine the percentage of apoptotic and necrotic cell death as described for panel **(A)** Transcription level expression of cell survival associated genes *BAX* and *HSP27* in α-hGM-CSF antibody (2 μg/10^6^ macrophages)-treated and untreated macrophages with and without *M. tuberculosis* infection was measured at seven days post-treatment/infection *via* qPCR assay. **(D)** ROS and NO levels in α-hGM-CSF antibody-treated and untreated (IgG isotype-treated) macrophages at day 7 post-*M. tuberculosis* infection, as measured through DCFDA and DAF2-DA probes, respectively, using a fluorimeter. Effect of nitric oxide synthase inhibitor L-NMMA (2 μM/10^6^ macrophages) on bacterial burden **(E)** and host cell viability **(F)** of *M. tuberculosis*-infected macrophages over seven days as measured through CFU and alamarBlue assays, respectively. **(G)** Intracellular levels of antimicrobial peptide LL-37 in α-hGM-CSF -treated and untreated macrophages as measured *via* ELISA at seven days post-infection. **(H)** Levels of LC3B puncta in α-hGM-CSF antibody-treated and untreated macrophages in the presence and absence of L-NMMA (2 μM/10^6^ macrophages) treatment at 7 days post-*M. tuberculosis* infection. LC3B positive puncta were detected through LC3B antibody staining using a fluorescent microscope. Effect of autophagy inducer (rapamycin) and autophagy inhibitor (3-MA) on bacterial burden **(I)** and host cell viability **(J)** of *M. tuberculosis*-infected macrophages over seven days as measured by CFU and alamarBlue assays, respectively. Treatment with hGM-CSF or α-hGM-CSF antibody/IgG isotype was performed every seven days beginning from the day of infection. Mtb, *M. tuberculosis*; pgs, picograms. Data represent average of three independent experiments carried out in duplicate. Bars and error bars represent means and SD, respectively. *p ≤ 0.05, **p ≤ 0.005.

The molecular mechanism through which GM-CSF may regulate host cell survival and bacterial containment is unknown. We investigated the downstream effects of GM-CSF supplementation/depletion on cell death and survival pathways in uninfected and *M. tuberculosis*-infected cells. Specifically, we assessed transcriptional expression of cell death/survival related genes *HSP27, BCL-xL, BCL-2, BAX/BAK*, and *MCL1* and the mode of cell death during exogenous hGM-CSF supplementation/antibody-mediated depletion. *M. tuberculosis*-infected macrophages supplemented with exogenous hGM-CSF (2 ng/1 million cells) showed significantly less apoptotic cell death over 14 days relative to untreated macrophages (**Figure 4A**). Moreover, apoptotic cell death was more common than necrotic cell death in infected macrophages. GM-CSF supplementation resulted in significantly lower levels of apoptosis even in uninfected macrophages (**Supplementary Figure S3**), indicating a homeostatic role for GM-CSF in preventing apoptotic cell death. Anti-apoptotic gene *BCL-2* was upregulated ~2-fold in hGM-CSF-treated macrophages in infected and uninfected groups at day 7 (**Figure 4B**). However, *BCL-xL*, another anti-apoptotic gene, did not vary between groups. Pro-apoptotic gene *BAX* was not significantly up or downregulated in hGM-CSF-treated macrophages relative to untreated controls. Apoptosis as well as necrosis significantly increased in infected and uninfected macrophages treated with α-hGM-CSF antibodies, and we observed a corresponding increase in pro-apoptotic *BAX* expression (**Figure 4C**). Cell survival-associated gene *HSP27* was downregulated significantly in *M. tuberculosis*-infected macrophages treated with α−hGM-CSF antibody; uninfected macrophages showed a similar profile with comparatively lower magnitude of difference.

Together, these data indicated a pattern of apoptosis and necrosis inhibition in macrophages with higher GM-CSF levels that were most prominent in infected macrophages. To determine whether this GM-CSF effect was mediated by apoptosis inhibition or other antimicrobial host defense pathways, we assessed antimicrobial effector mechanisms, including reactive oxygen species (ROS)/nitric oxide (NO) generation, antimicrobial peptide production, autophagic flux in GM-CSF-competent and GM-CSF-depleted macrophages during *M. tuberculosis* infection. GM-CSF-competent macrophages did not show any significant differences in ROS production levels compared to GM-CSF-depleted macrophages (**Figure 4D**). NO levels in GM-CSF-competent macrophages were significantly higher compared to GM-CSF-depleted macrophages, indicating that NO may be an antimicrobial effector mechanism induced by GM-CSF signaling. To determine if an NO-mediated effector mechanism was associated with macrophage survival, we measured host and bacterial cell survival in GM-CSF-competent and GM-CSF-depleted macrophages treated with a pharmacological inhibitor of NO; NG-Methyl-L-arginine acetate salt (L-NMMA) (**Figures 4E, F**). In GM-CSF-competent macrophages, L-NMMA treatment resulted in increased bacterial burden but not significant cell death. In GM-CSF-depleted macrophages, untreated and L-NMMA-treated cells showed increased bacterial burden and host cell death. These data suggest that NO contributes to GM-CSF-mediated antimicrobial effector mechanisms but not host cell survival.

Since NO is known to induce production of antimicrobial peptides and activate autophagy, which could also contribute to boosting intracellular host defenses, antimicrobial peptide cathilicidin (LL-37) and autophagic flux in GM-CSF competent and depleted macrophages were further examined. LL-37 levels were similar between GM-CSF-competent and GM-CSF-depleted macrophages, indicating that GM-CSF-mediated restriction of *M. tuberculosis* bacilli in macrophages is not dependent on LL-37 (**Figure 4G**). Strikingly, increased autophagy flux (as examined through the LC3B positive puncta) in GM-CSF competent macrophages was observed as compared to GM-CSF depleted macrophages (**Figure 4H**). Increased LC3 B gene expression at the transcription level was also observed in hGM-CSF-treated infected and uninfected macrophages relative to untreated macrophages (**Supplementary Figure S4**). Interestingly, blockade of NO *via* L-NMMA did not affect autophagic flux in GM-CSF competent/depleted macrophages, indicating that GM-CSF-mediated induction of autophagy is NO-independent (**Figure 4H**). To determine whether GM-CSF-mediated autophagy affects host and bacterial cell survival, we induced and blocked autophagy using rapamycin and 3-MA, respectively, in GM-CSF-competent macrophages during *M. tuberculosis* infection. Low-dose rapamycin resulted in decreased bacterial burden, but higher doses reversed this effect. Blockade of autophagy through 3-MA resulted in increased bacterial burden and decreased cell survival (**Figures 4I, J**). Thus, GM-CSF-dependent host cell survival and antimicrobial effector mechanisms may be partially autophagy-dependent; however, the data also indicated that precise regulation of autophagy may be equally important for pathogen restriction and host cell survival.

### GM-CSF Mediates Regulatory Immune Responses in Macrophages and Increases Antigen Presentation to T Cells During *M. tuberculosis* Infection

**Figure 5:**
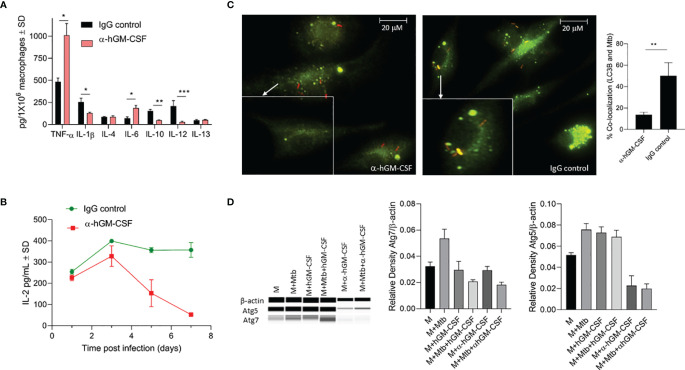
Effect of α-hGM-CSF antibody on pro- and anti-inflammatory cytokine secretion, antigen presentation, and phagosome maturation of macrophages. **(A)** Secreted levels of pro- and anti-inflammatory cytokines by GM-CSF-competent (control) and GM-CSF-depleted (α-hGM-CSF antibody-treated) macrophages at three days post-infection with *M. tuberculosis*. MOI = 1; α-hGM-CSF antibody = 2 μg/10^6^ macrophages. **(B)** Secreted levels of IL-2 by *M. tuberculosis* Ag85B-specific T cells during co-culture with GM-CSF competent (control) and GM-CSF depleted (α-GM-CSF antibody-treated) macrophages at different times post-infection. **(C)** Localization of *M. tuberculosis* (red) phagosomes and LC3B+ autophagosomes (green) within GM-CSF competent (control) and GM-CSF depleted (α-GM-CSF antibody-treated) macrophages after seven days of infection. Phagosomes were labeled using RFP *M. tuberculosis* and autophagosomes were labeled with LC3B-specific antibodies. Images were acquired at 60x magnification with an inverted fluorescent microscope using macrophages of a single random healthy donor and were analyzed using confocal microscopy and NIKON NIS element software. Bar graph shows the quantitation of percent co-localization of *M. tuberculosis* and autophagosomes in macrophages representing an average of three replicates, each including 25 fields. **(D)** Western blot profile of Atg5, Atg7, and β- actin expression by macrophages treated with hGM-CSF/α-hGM-CSF antibody relative to untreated macrophages three days with and without *M. tuberculosis* infection (three days post-infection). M, macrophages; Mtb, *M. tuberculosis*; pg, picograms. Data are representative of three independent experiments carried out in duplicate. Bars and error bars represent means and SD, respectively. *p ≤ 0.05, **p ≤ 0.005, ***p ≤ 0.0005.

We evaluated a panel of pro- and anti-inflammatory cytokines in GM-CSF-competent/deficient macrophages three days post-*M. tuberculosis* infection (**Figure 5A**). GM-CSF-depleted macrophages secreted significantly lower levels of IL-1β, IL-12, and IL-10 and significantly higher levels of TNF-α and IL-6 compared to GM-CSF-competent macrophages. No significant difference was observed in IL-4 and IL-13 levels. Thus, GM-CSF signaling may simultaneously be inducing pro-and anti-inflammatory immune responses and a dual GM-CSF response may be considered more of a regulatory immune response than a pro-inflammatory response. Interestingly, IL-12 levels from GM-CSF-competent macrophages were high relative to GM-CSF-deficient macrophages, suggesting that GM-CSF may induce macrophage maturation (22).

We next evaluated whether GM-CSF-competent/deficient macrophages had different T cell priming capabilities during *M. tuberculosis* infection. We used an *in vitro* antigen presentation assay using a CD4 hybridoma T cell recognizing a specific epitope on Ag85B to assess T cell priming by GM-CSF-competent/deficient macrophages. IL-2 levels secreted from hybridoma T cells were monitored over time with overlay to *M. tuberculosis*-infected macrophages (**Figure 5B**). GM-CSF-competent macrophages induced higher levels of antigen presentation after two days of infection, though the difference was not significant relative to GM-CSF-depleted macrophages. However, GM-CSF competent macrophages were able to sustain a much higher levels of antigen presentation beyond day 3 post infection as compared to GM-CSF deficient macrophages, hence further suggesting the critical role of GM-CSF signaling in sustenance of antigen presentation and efficient processing and presentation of antigens to CD4T cells. it is also possible that low number of viable cells in GM-CSF deficient macrophages could have also contributed to the reduction in overall levels of antigen presentation. However, the magnitude of difference in IL-2 levels secreted by untreated vs anti-GM-CSF antibody treated macrophages is much higher at day 7 post infection (almost 10 fold: 30 picograms vs 300 picograms) (**Figure 5B**) as compared to the magnitude of difference in cell viability of untreated vs anti-GM-CSF antibody treated macrophages (less than 2 fold) (**Figure 3E**) at day 7 post infection. We thus believe that during the late phase of infection, GM-CSF is affecting the antigen presentation capability of macrophages.

We further compared phagosome maturation events in GM-CSF-competent/deficient macrophages after *M. tuberculosis* infection. At three days post-infection, over 50% of *M. tuberculosis*-containing phagosomes colocalized with LC3B positive endosomes in GM-CSF-competent macrophages, with GM-CSF deficient macrophages exhibiting less than 20% co-localization (**Figure 5C**). Notably, this difference was not significant during the first 24 h of infection (data not shown). Thus, increased fusion of LC3B containing endosomes with *M. tuberculosis* bacilli indicated an autophagy dependent maturation of phagosomes. Autophagosome-associated LC3B and other autophagy-related genes, including LC3B, Atg5 and Atg7, also demonstrated increased expression at the transcription/translational level in hGM-CSF-treated macrophages and decreased expression in α-GM-CSF-treated macrophages (**Figures 5C, D**), further indicating the role of GM-CSF in macrophage autophagy regulation. Thus, increased maturation of *M. tuberculosis*-containing phagosomes *via* GM-CSF-mediated autophagy may degrade *M. tuberculosis* bacilli and process its antigens, resulting in improved and sustained antigen presentation in macrophages.

### Macrophages From Active TB Patients Produce Lower Levels of GM-CSF and Exhibit a Distinct Metabolic State

**Figure 6:**
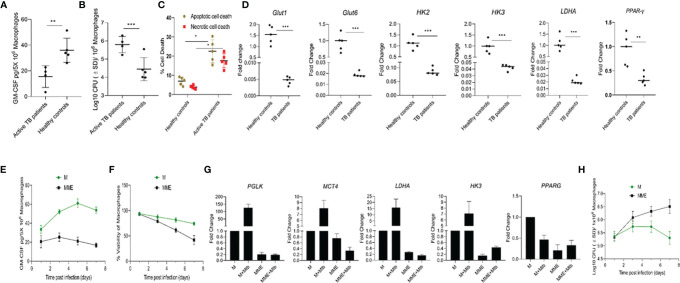
Different levels of GM-CSF production in macrophages of active TB patients and healthy controls and its association with intracellular bacterial control, survival of the host cells, and metabolic state. **(A)** Secreted levels of GM-CSF by macrophages of active TB patients versus healthy controls post-differentiation and *M. tuberculosis* infection. GM-CSF levels were measured at day seven post-infection. **(B)** *M. tuberculosis* burden in macrophages of active TB patients versus healthy controls post-differentiation measured by CFU assay at seven days after infection. **(C)** Percentage and proportion of apoptotic and necrotic cell death in *M. tuberculosis*-infected macrophages of active TB patients versus healthy controls at seven days post-infection measured by fluorescent microscopy-based apoptotic/necrotic assay. **(D)** Relative fold change in RNA concentration of specific genes (*GLUT1, GLUT6, HK2, HK3, LDHA*, and *PPAR-γ*) in PBMC-derived macrophages of active TB patients versus healthy subjects as determined by qPCR. Student’s t-test was used for means comparison between active TB patients versus healthy subjects. **(E)** Secreted levels of GM-CSF by M versus MME macrophages at different times post-*M. tuberculosis* infection. GM-CSF levels were measured in macrophage (5x10^6^) culture supernatants using sandwich ELISA. **(F)** Cell viability of *M. tuberculosis*-infected M versus MME macrophages measured by alamarBlue assay over seven days of infection. **(G)** Relative fold change in RNA concentration of specific genes (*PGLK, MCT4, LDHA, HK3*, and *PPAR-γ*) in PBMC-derived macrophages of active TB patients versus healthy subjects as determined by qPCR. Student’s t-test was used for means comparison between M versus MME macrophages. **(H)** *M. tuberculosis* burden in M versus MME macrophages as measured through CFU assay over seven days of infection. Mtb, *M. tuberculosis*; pgs, picograms. Data represent the average of three independent experiments carried out in duplicate. Bars and error bars represent means and SD, respectively. *p ≤ 0.05, **p ≤ 0.005, ***p ≤ 0.0005.

As our results indicated that natural variations in GM-CSF production/signaling could be a factor in determining an individual’s vulnerability to TB, we conducted parallel studies to compare patterns of GM-CSF production in macrophages from individuals with active TB to healthy controls. Monocytes from healthy controls and newly diagnosed TB patients were isolated and compared for their ability to produce GM-CSF, control host cell survival, and restrict *M. tuberculosis* growth. *M. tuberculosis* infection increased macrophage GM-CSF production in both populations after seven days of infection (**Figure 6A**), though macrophages from active TB patients (n=4) produced significantly less than those from healthy controls (n=5). These levels remained low throughout the 14-day infection period (**Supplementary Figure S5**) and translated to increased bacterial burden and host cell death relative to healthy controls (**Figures 6B, C**).

Because some of our active TB population also had preexisting diabetic conditions, which can cause a metabolic reprogramming of immune cells that leads to defective immune response against TB, we examined whether diabetes-induced metabolic changes were associated with macrophage GM-CSF production. We first characterized the central metabolic state of macrophages from active TB patients and healthy controls by examining the expression of genes related to glycolysis and oxidative phosphorylation. Macrophages from active TB patients exhibited significant downregulation (1.5- 20 fold) of genes encoding glucose transporters 1 and 6 (GLUT1 and 6), glycolytic enzymes/isozymes hexokinases (HK2 and HK3), phosphofructokinase 1 (PFK1), and lactate dehydrogenase A (LDHA) relative to healthy controls (**Figure 6D**). Further, decreased glycolytic flux also resulted in concurrent downregulation of lipid metabolism-related gene Peroxisome Proliferator-Activated Receptor Gamma (PPAR-γ) in macrophages from active TB patients relative to controls.

We next evaluated whether metabolic dysregulation was causing reduced GM-CSF production in macrophages. We induced metabolic dysregulation in macrophages by treating cells with glucose, insulin, and palmitate (23) and quantified GM-CSF production in these metabolically dysregulated macrophages (MME macrophages) relative to a control group of untreated cells from the same donor. MME macrophages produced significantly reduced levels of GM-CSF, and exhibited significantly reduced survival following *M. tuberculosis* infection relative to untreated control macrophages (**Figures 6E, F**). We also observed reduced expression of glycolysis-related genes Protein glycosylation K (*PGLK)*, Monocarboxylate transporter 4 *(MCT4), LDHA*, and *HK3*, and lipid metabolism-related gene *PPAR-γ *in MME macrophages relative to control macrophages (**Figure 6G**). MME macrophages also showed a significantly higher intracellular *M. tuberculosis* burden relative to untreated macrophages (**Figure 6H**). Thus, metabolic dysregulation may also cause a reduction in macrophage GM-CSF production that results in their reduced survival and increased permissiveness to *M. tuberculosis* growth.

## Discussion

In humans, the development of adaptive immunity against *M. tuberculosis* infection takes 2-3 weeks after first exposure (24). However, symptomatic disease does not appear in 90% of *M. tuberculosis*-infected individuals during this early stage when mostly innate immune cells are participating in the immune response. This indicates initial clearance or containment of infection by the innate immune system, and highlights the importance of innate immunity in controlling *M. tuberculosis* infection. Macrophages are both the primary host cells for *M. tuberculosis* and the first line of innate cellular defense (25). Macrophage populations that interact with *M. tuberculosis* have exhibited tremendous heterogeneity and plasticity, with diverse functional phenotypes. The local microenvironment in which *M. tuberculosis* interacts with macrophages has been suggested to influence the phenotype of macrophages and the outcome of infection (26, 27). However, the secreted soluble mediators and/or other immune components that constitute the microenvironment of macrophages during *M. tuberculosis* infection are not understood well.

GM-CSF is one of the cytokines that may polarize macrophages toward the protective phenotype and has been increasingly recognized as an essential factor for TB resistance (13, 28, 29). A recent study showed that GM-CSF is produced upon infection with *M. tuberculosis* and that human macrophages’ antimycobacterial properties correlated with their ability to produce GM-CSF (14). In a mouse model of TB, we and others have previously shown the GM-CSF expression in lung epithelial cells and the hematopoietic compartment (16, 30, 31). More recent investigations have shown that conventional and non-conventional T cells (iNK and γδ T cells) also produce GM-CSF, which was critical for host protection during experimental TB in mice (32). However, a key observation often overlooked is that the manifestation of TB pathogenesis and overall outcome of infection in humans is different than in mice, which are naturally susceptible to TB. Therefore, it is imperative to examine whether differential GM-CSF production and its association with TB susceptibility/resistance exists in the human population.

The study presented here focused only on macrophage-produced GM-CSF and suggests GM-CSF’s critical role in controlling infection and preventing cell death of macrophages (**Figures 1**, **2**). However, it does not rule out the possible contribution of other myeloid and non-myeloid cell-produced GM-CSF in protective immunity against TB. One study in a mouse model of TB found that conventional T cell- (CD4 and CD8) produced GM-CSF was also protective and required for control of *M. tuberculosis* infection, though only when GM-CSF is not produced by other cell types (32). In the current study, the human macrophages that were able to produce competent levels of GM-CSF contained the *M. tuberculosis* infection (**Figure 1C**) even in the absence of T cells, indicating that T cell-produced GM-CSF may not be essential in humans if macrophages can produce the required quantity of GM-CSF. Nonetheless, it is possible that T cell-produced GM-CSF could be important when macrophages or other cells are not producing enough. Contribution of non-conventional/conventional T cell-produced GM-CSF in controlling TB in a mouse model of TB could also be due to sub-optimal production of this cytokine by their macrophages. Indeed, a recent study confirmed that mouse macrophages produced significantly less GM-CSF compared to human macrophages after infection with *M. tuberculosis* (17); this directly correlated with their reduced life span and ability to control *M. tuberculosis* infection. In our study, differentiated macrophages in the majority of our human samples were able to control *M. tuberculosis* growth and survive for an extended period, as seen in the majority of humans who do not develop active TB disease after exposure (**Figure 1**).

The number of *M. tuberculosis* bacilli in differentiated macrophages in most individuals in our study remained restricted and decreased over a period of time (**Figure 1B**). Interestingly, when monocytes were infected with *M. tuberculosis* prior to their differentiation, increased intracellular growth of the pathogen was observed as well as accelerated death of host cells (**Figures 1F, G**). At the same time, these infected undifferentiated monocytes were also unable to produce GM-CSF, indicating that *M. tuberculosis* can interfere with monocytes’ natural ability to produce GM-CSF and differentiation into macrophages. In contrast, GM-CSF production was increased in differentiated macrophages after *M. tuberculosis* infection (**Figure 1A**). This up-regulation of GM-CSF by *M. tuberculosis* has been shown to be mediated by the PI3-K/MEK1/p38 MAPK-associated signaling pathway (33). However, it is unclear how this phenomenon is reversed in undifferentiated macrophages. There is considerable evidence of increased permissiveness of undifferentiated monocytes to *M. tuberculosis* growth. Chronic inflammatory response is known to drive this phenomenon that eventually results in increased host cell death (34). An increased bacterial burden and associated cell death of undifferentiated monocytes post-*M. tuberculosis* infection (**Figures 1F, G**) corroborated these earlier findings and further indicated that cell death triggered by *M. tuberculosis* could have caused the reduction in secreted GM-CSF levels by undifferentiated monocytes.

While increased infection-induced cell death was observed in undifferentiated monocytes, differentiated macrophages were able to prevent cell death after *M. tuberculosis* infection (**Figures 2B, C, E, F**). Human TB granulomas are also known to express GM-CSF (35). The protective effect of this cytokine against TB in mediating granulomatous response and restriction of the pathogen within macrophages has been shown previously (16, 36). We sought to determine the mechanism through which GM-CSF could have prevented infection-induced cell death. Anti-apoptotic genes, as well as self-renewal genes such as *BCL-2*, were upregulated in GM-CSF-competent macrophages (**Figure 4B**). GM-CSF is a known mitogenic signal for the local proliferation and self-renewal of macrophages which indicates that GM-CSF may increase the survival of the host cells by enhancing activation of cell proliferation genes. Indeed, local proliferation of macrophages has been described in the granulomatous lesions formed during experimental Goodpasture’s syndrome (37, 38), similar to the granulomatous response and proliferative behavior we observed in GM-CSF-competent macrophages (**Figure 3A**). Morover, enhanced cell proliferation of macrophages during granuloma formation is also known to reduce apoptosis (39). Thus, GM-CSF-mediated self-renewal/survival, local proliferation of macrophages in the lungs, and increased formation of protective granulomatous structures during TB accentuate the importance of granuloma targeting therapies for TB.

Intracellular host defense mechanisms triggered by GM-CSF are poorly understood. We used α-hGM-CSF antibody to identify the involvement of these antimicrobial effector mechanisms (**Figures 4E, F, H**). We demonstrated that GM-CSF-mediated regulation of autophagy was not only critical in controlling the bacterial burden within macrophages, but also in preventing cell death (**Figures 4I, J**). While excessive exogenous supplementation of GM-CSF resulted in macrophage cell death, controlled supplementation of this cytokine enhanced macrophage cell survival as well as bacterial killing (**Figures 2E, F**, and **3C**). These data indicate the critical role of a precisely regulated autophagic process in protecting against *M. tuberculosis* infection, which has been previously documented (40). GM-CSF-competent macrophages also exhibited lower levels of inflammatory cytokines and higher levels of autophagic flux as compared to GM-CSF-deficient macrophages (**Figure 5**), suggesting the possible role of GM-CSF-mediated autophagy in the reduction of inflammation during *M. tuberculosis* infection (41). Along this line, further research defining the mechanistic basis of anti-inflammatory as well as anti-apoptotic effect of autophagic cellular processes during *M. tuberculosis* infection are warranted to fully realize the potential of GM-CSF targeted host directed therapies.

Remarkably, simultaneous production of pro- and anti-inflammatory cytokines by GM-CSF-competent macrophages restricted intracellular *M. tuberculosis* infection (**Figure 5A**) which further highlights the importance of a balanced immune response to effectively control infection without causing damage. For instance, GM-CSF-competent macrophages produced significantly more IL-12 but less TNF-α compared with GM-CSF-deficient macrophages (**Figure 5A**), even though both of these cytokines are pro-inflammatory. TNF-α is a known mediator of tissue damage during TB (42). Thus, downregulation of TNF-α could be useful in reducing the cellular damage caused by TB. IL-12 mediates early T cell activation without causing excessive inflammation during *M. tuberculosis* infection (43). IL-12 also induces maturation of macrophages and dendritic cells, which is critical for effective antigen presentation to activate antigen specific T cells during *M. tuberculosis* infection (44, 45). Improved antigen presentation was indeed evident in GM-CSF-competent macrophages that produced higher levels of IL-12 (**Figures 5A, B**). This IL-12-mediated induction of antigen presentation in antigen presenting cells (APCs) could help in generating optimal interferon-γ (IFN-γ) response by T cells, which can further contribute to the restriction of *M. tuberculosis* growth in macrophages/dendritic cells. Nonetheless, the mechanism through which GM-CSF promotes antigen presentation along with the cellular processes that manifest the dual control of both inflammatory as well as anti-inflammatory cytokines in APCs needs to be fully understood to ascertain the therapeutic potential of GM-CSF against TB.

GM-CSF-mediated control of immune response in macrophages also appears interconnected with the regulation of cellular metabolism. *M. tuberculosis* is known to remodel the metabolism of immune cells and it has been proposed that the net outcome to *M. tuberculosis* infection clearance or chronic disease is likely dependent on the combined immunologic and metabolic status of the immune cells that participate in the infection process (46). GM-CSF-competent macrophages did not allow skewing of bioenergetics toward the glycolysis and pentose phosphate pathway after *M. tuberculosis* infection (**Figure 6D**). Thus, GM-CSF could play a key role in resisting the metabolic changes induced by *M. tuberculosis* infection and maintain a homeostatic metabolic status in phagocytes. Resistance to the metabolic reprogramming of macrophages during *M. tuberculosis* infection can protect their effector functions and antimicrobial response, which can ultimately govern the outcome of *M. tuberculosis*-host interactions. Active TB patient macrophages with decreased GM-CSF production displayed increased susceptibility to *M. tuberculosis*, further highlighting the link between dysregulation of cellular metabolism and GM-CSF (**Figures 6A–D**). While macrophages of active TB patients who had preexisting DM produced less GM-CSF compared to healthy controls, our data indicate that metabolic dysregulation is likely responsible for the reduced level of GM-CSF (**Figures 6E–G**). However, more work is needed to elucidate the intricate relationship between metabolic programming pathways and GM-CSF signaling.

A comprehensive understanding of the immune-metabolic processes occurring in host cells during *M. tuberculosis* infection, and the host/bacterial components that mediate their regulation will drive novel treatment/vaccination strategies to control TB more effectively. With future studies, the GM-CSF signaling pathway that regulates innate immune responses and cellular metabolism could be harnessed for host-directed therapeutic strategies to promote bacterial clearance and improve treatment outcomes.

## Data Availability Statement

The raw data supporting the conclusions of this article will be made available by the authors, without undue reservation.

## Ethics Statement

The studies involving human participants were reviewed and approved by Institutional Review board, University of Texas Health Science center. The patients/participants provided their written informed consent to participate in this study.

## Author Contributions

AK designed the study, conducted experiments and analyzed the data; AM and VS designed and conducted experiments and analyzed the data; BR provided specimens and analyzed the data. AK wrote the manuscript, SS, CJ, MG, and BR provided feedback on the manuscript. All authors contributed to the article and approved the submitted version.

## Conflict of Interest

The authors declare that the research was conducted in the absence of any commercial or financial relationships that could be construed as a potential conflict of interest.

## Publisher’s Note

All claims expressed in this article are solely those of the authors and do not necessarily represent those of their affiliated organizations, or those of the publisher, the editors and the reviewers. Any product that may be evaluated in this article, or claim that may be made by its manufacturer, is not guaranteed or endorsed by the publisher.

## Abbreviations

TB, Tuberculosis; GM-CSF, Granulocyte Macrophage Colony Stimulating Factor; TNF-α, Tumor Necrosis Factor alpha; IL-1β, Interluking 1 beta; IL-6, Interleukin 6; Il-10, Interleukin 10; IL-12, Interleukin 12; IL-13, Interleukin 13; BCL-2, B Cell Lymphoma 2; HSP27, Heat Shock Protein 27; IFN-γ, Interferon gamma; HLA-DR, Human Leukocyte Antigen – DR isotype; NO, Nitric Oxide; DM, Diabetes Mellitus; PI3K, Phosphoinositide 3-Kinase; MEK1, Mitogen-Activated Protein Pinase 1; p38 MAPK, p38 Mitogen-Activated Protein Kinases; Ink T cell, Invariant Natural Killer T cells; CD4, Cluster of Differentiation 4; APC, Antigen Presenting Cells.
