# Supplementary material for: Human Macrophages Exhibit GM-CSF Dependent Restriction of Mycobacterium tuberculosis Infection via Regulating Their Self-Survival, Differentiation and Metabolism
Source: Front Immunol. 2022 May 12;13:859116. doi: 10.3389/fimmu.2022.859116 (PMC9134823; doi:10.3389/fimmu.2022.859116)
Supplement: Supplementary file 1 [file DataSheet_1.docx]

***SUPPLEMENTARY FIGURES: Human macrophages exhibit GM-CSF dependent restriction of Mycobacterium tuberculosis infection via regulating their self-survival, differentiation and metabolism***


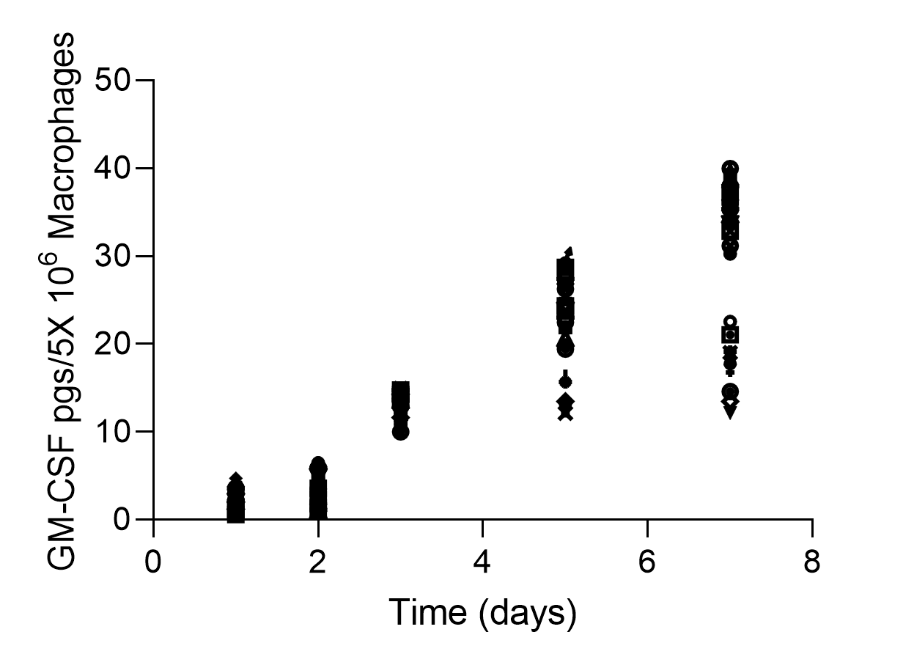


**S1:** Secreted levels of GM-CSF by monocytes of different individuals during their differentiation. GM-CSF levels were measured in the supernatants of macrophage (5x10^6^) culture by sandwich ELISA method (pgs = picograms). Data represent the average of three independent experiments carried out in duplicate.


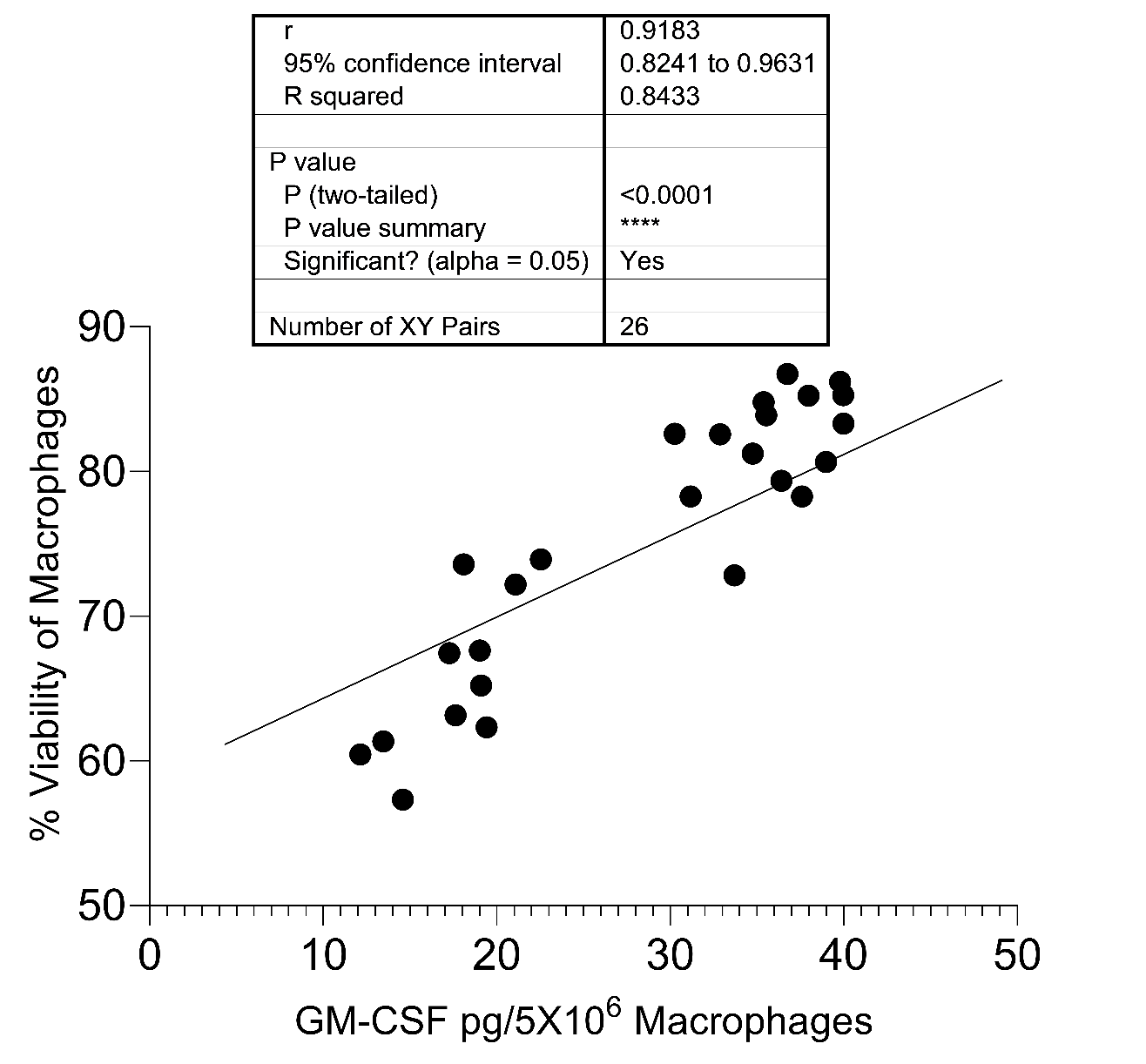


**S2:** Two tailed correlation analysis between secreted GM-CSF levels and cell viability of uninfected macrophages of different individuals at day 7 post differentiation. Data represent the average of three independent experiments carried out in duplicate.


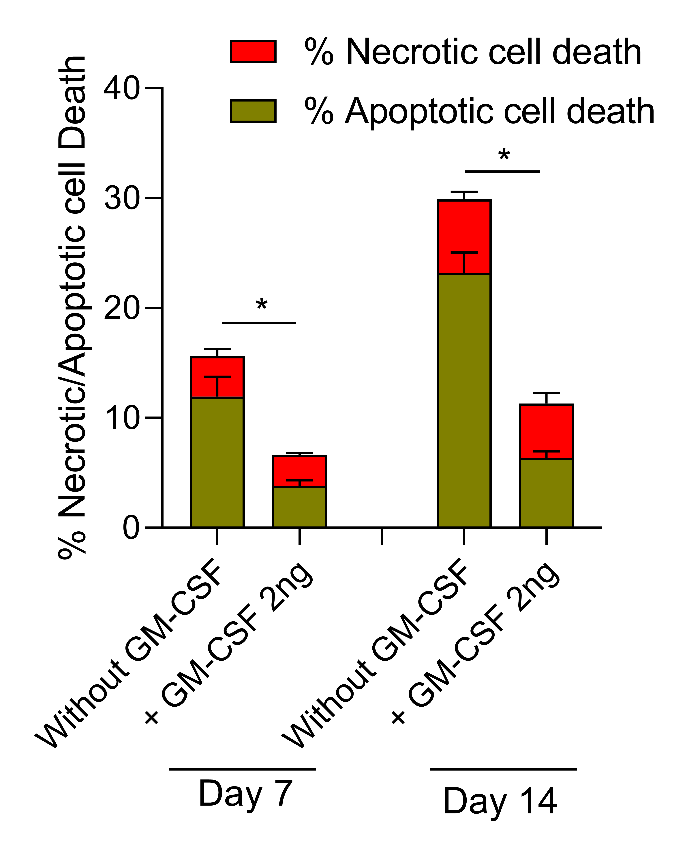


**S3:** Percentage and proportion of apoptotic and necrotic cell death after 7 and 14 days post-differentiation in untreated and GM-CSF (2 ng/10^6^ macrophages)-treated macrophages. At 7 and 14 days post-differentiation, GM-CSF-treated and untreated macrophages were incubated with Apopxin solution and 7-ADD, and fluorescence [Ex/Em=490/525 nm (apoptosis, Ex/Em=550/650 nm (necrosis), Ex/Em=405/450 nm (healthy cells) was measured by fluorescence microscopy at a magnification of 20X counting 25 fields per replicate for each condition. Treatment with human GM-CSF was done once every seven days beginning from the day of infection. Data represent average of three independent experiments carried out in duplicate. Bars and error bars represent means and SD, respectively. *p≤0.05, **p≤0.005, ***p≤0.0005, ****p≤0.0001.


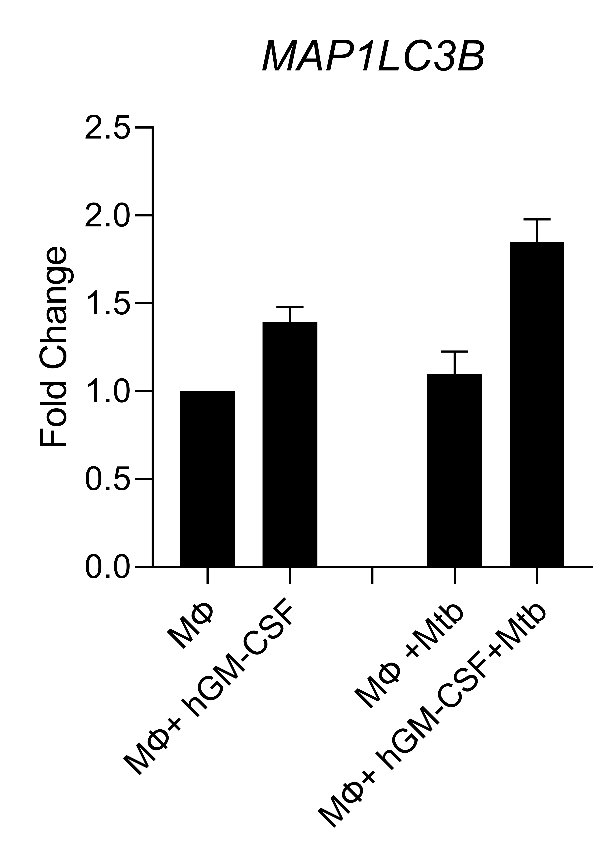


**S4:** Transcription level expression of autophagy associated gene *MAP1LC3B* genes in GM-CSF (2 ng/10^6^ macrophages)-treated and untreated macrophages with and without *M. tuberculosis* infection. Gene expression was measured at seven days post-treatment/infection via qPCR assay. Mφ= Macrophage. Data represent average of three independent experiments carried out in duplicate. Bars and error bars represent means and SD, respectively. *p≤0.05, **p≤0.005, ***p≤0.0005, ****p≤0.0001.


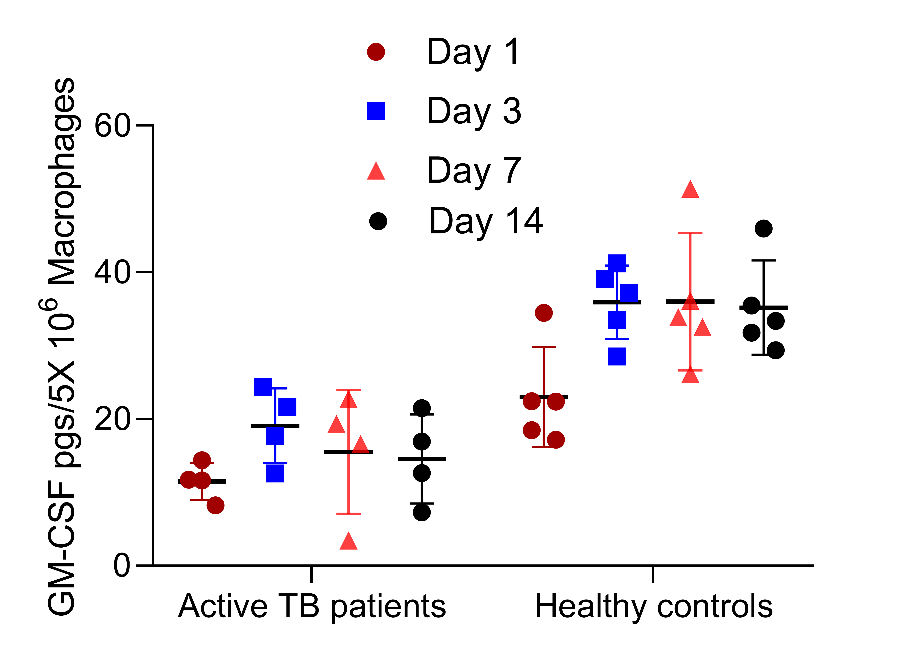


**S5:** Secreted levels of GM-CSF by macrophages of active TB patients versus healthy controls at different time post-differentiation. Data represent the average of three independent experiments carried out in duplicate. Bars and error bars represent means and SD, respectively. *p≤0.05, **p≤0.005, ***p≤0.0005, ****p≤0.0001.

**Supplementary Table 1:** % Viability of macrophages isolated from different healthy donors at 7 days post *M. tuberculosis* infection.

| Donor ID | % Viability of macrophages (day 7 post *M. tuberculosis* infection |
| --- | --- |
| D1 | 92.59 |
| D2 | 90.65 |
| D3 | 93.29 |
| D4 | 70.12 |
| D5 | 83.03 |
| D6 | 84.35 |
| D7 | 84.22 |
| D8 | 80.41 |
| D9 | 82.93 |
| D10 | 73.90 |
| D11 | 64.16 |
| D12 | 93.01 |
| D13 | 92.16 |
| D14 | 80.64 |
| D15 | 68.37 |
| D16 | 92.40 |
| D17 | 90.75 |
| D18 | 91.14 |
| D19 | 92.73 |
| D20 | 73.64 |
| D21 | 77.08 |
| D22 | 82.32 |
| D23 | 90.08 |
| D24 | 83.14 |
| D25 | 87.39 |
| D26 | 88.49 |

**Supplementary table 2:** Primers used for qPCR analysis of different genes expressed at transcriptional level.

| Gene | Forward primer 5'->3' | Reverse primer 5'->3' |
| --- | --- | --- |
| GAPDH | GGAGCGAGATCCCTCCAAAAT | GGCTGTTGTCATACTTCTCATGG |
| BAX | GGTTGTCGCCCTTTTCTA | CGGAGGAAGTCCAATGTC |
| BCL2 | TCCTTGTCTACGCTTTCCACG | GGTCGCATTGTGGCCTTT |
| BCL-XL | TCCTTGTCTACGCTTTCCACG | GGTCGCATTGTGGCCTTT |
| GLUT1 | TTGCAGGCTTCTCCAACTGGAC | CAGAACCAGGAGCACAGTGAAG |
| GLUT6 | TTGCAGGCTTCTCCAACTGGAC | CAGAACCAGGAGCACAGTGAAG |
| HK2 | GAGTTTGACCTGGATGTGGTTGC | CCTCCATGTAGCAGGCATTGCT |
| HK3 | CATCGTGGACTTCCAGCAGAAG | CTTGGTCCAGTTCAGGAGGATG |
| HSP27 | TCCCTGGATGTCAACCACTTC | TCTCCACCACGCCATCCT |
| LDHA | GGATCTCCAACATGGCAGCCTT | AGACGGCTTTCTCCCTCTTGCT |
| MCT4 | GCCATCTTTGCTGGTGGTTACC | TGGTCCAGAAAGGACAGCCATC |
| PGLK | CCGCTTTCATGTGGAGGAAGAAG | CTCTGTGAGCAGTGCCAAAAGC |
| PPARγ  MAP1LC3B | CGTGGCCGCAGATTTGAA  GAGAAGCAGCTTCCTGTTCTGG | CTTCCATTACGGAGAGATCCAC  GTGTCCGTTCACCAACAGGAAG |
